# Can we throw information out of visual working memory and does this leave informational residue in long-term memory?

**DOI:** 10.3389/fpsyg.2014.00294

**Published:** 2014-04-08

**Authors:** Ashleigh M. Maxcey, Geoffrey F. Woodman

**Affiliations:** ^1^Department of Psychology, Manchester UniversityNorth Manchester, IN, USA; ^2^Vanderbilt Vision Research Center and Center for Integrative and Cognition Neuroscience, Department of Psychology, Vanderbilt UniversityNashville, TN, USA

**Keywords:** purging, process purity, directed-forgetting, visual working memory, long-term memory

## Abstract

Can we entirely erase a temporary memory representation from mind? This question has been addressed in several recent studies that tested the specific hypothesis that a representation can be erased from visual working memory based on a cue that indicated that the representation was no longer necessary for the task. In addition to behavioral results that are consistent with the idea that we can throw information out of visual working memory, recent neurophysiological recordings support this proposal. However, given the infinite capacity of long-term memory, it is unclear whether throwing a representation out of visual working memory really removes its effects on memory entirely. In this paper, we advocate for an approach that examines our ability to erase memory representations from working memory, as well as possible traces that those erased representations leave in long-term memory.

Many science fiction plots involve the erasure of memory (Dick, 1966; Rowling, 2000), yet surprisingly little is known about the plausibility of erasing information from memory. Recent evidence indicates that information can indeed be erased from working memory (part of this work referred to this operation as *purging*, but we will use erasing throughout this paper to be consistent; Williams and Woodman, 2012; Maxcey-Richard and Hollingworth, 2013). These empirical reports suggest that this seemingly futuristic process can be accomplished within our own minds with our onboard hardware and software. This is particularly noteworthy because the classic theoretical view of working memory is that information is only lost from this limited-capacity store through displacement by new information (e.g., Waugh and Norman, 1965). However, does this mean that we will have no memory for the information erased from working memory?

If we can completely erase representations from all memory stores, then this would have important real-world implications. For example, if we learn that a warning signal, a classmate, or an eyewitness has been providing unreliable information, then we could retroactively eliminate the information they had provided from memory. Indeed, people commonly act as though this is possible in the United States legal system. When a judge sustains an objection in the courtroom, she gives the jury instructions to disregard a line of questioning. However, findings from long-term memory studies have resulted in conflicting conclusions. One group of studies demonstrates the intentional erasure of information from long-term memory (Johnson, 1994; Baddeley et al., 2009), consistent with long-standing claims of benefits of such forgetting (Freud, 1946; Weiner, 1968; Anderson and Levy, 2009). In contrast, another group of studies suggests that erasing information from long-term memory might not typically occur (e.g., Bulevich et al., 2006; Storm et al., 2008, but see Anderson and Green, 2001), or at least is not necessary given the seemingly infinite capacity of long-term memory (Standing, 1973; Brady et al., 2008). Multistore models of memory propose that information passes through a capacity-limited working memory before reaching long-term memory if it is rehearsed in working memory for a sufficient period of time (e.g., Atkinson and Shiffrin, 1968). The erasure or replacement of information previously encoded into working memory could effectively wipe out those temporary memory representations (e.g., Ecker et al., 2010). But, this opens up the possibility that when we erase previously relevant information from working memory, a representation of that information may continue to linger in long-term memory.

Our goal in this paper is to review recent findings that purport to show that we can erase information from working memory while discussing possible explanations about what is going on in long-term memory during these paradigms. This will result in specific predictions and clearly advocate for neuroscientific methods to provide converging evidence that tests these predictions. We begin by discussing behavioral findings that suggest that subjects can erase working memory representations following cues that indicate certain memory representations are irrelevant to the task they are performing.

Maxcey-Richard and Hollingworth (2013) examined the top-down control of what information is maintained in visual working memory during online scene viewing. The basic paradigm they created simulated the real-world task of fixating objects sequentially. For example, if we search for an apple in the bin at the farmer’s market we begin by assigning priority to one of the objects (e.g., remembering the most appealing candidate apple). Then we maintain that representation across subsequent shifts of attention and the eyes (e.g., continuing the search and fixating other, less appealing apples). However, upon encountering a better candidate apple we need to flexibly reassign priority to representing that new object in memory.

In the laboratory task of Maxcey-Richard and Hollingworth (2013), subjects were shown a workshop scene to simulate the demands of this foraging task. Semantically appropriate objects (e.g., hammer, drill) sequentially appeared in ecologically valid locations throughout the scene and subjects were instructed to make a saccade to each new object when it appeared and the currently fixated object simultaneously disappeared. They were then instructed to maintain fixation on that object until a new object appeared. Each trial consisted of 6–10 objects drawn from a set of 10 paired token objects (e.g., two fire extinguishers, two screwdrivers). Eye movements were monitored with a camera-based eye tracker to ensure subjects followed instructions. Subjects were instructed that each trial would end with a token discrimination task, testing memory for an object that was present in the trial. In order to indicate which object should be prioritized during the trial, the appearance of each object was accompanied by an auditory tone. A high-pitched tone served as a cue indicating the object that was likely to be tested (e.g., simulating the best candidate apple). A low-pitched tone indicated an object was not likely to be tested (e.g., simulating the other less appealing apples). Critically, some trials included two high-pitched tones. Subjects were instructed that in the event of two high-pitched tones, the second cued object was the to-be-remembered object and the originally cued object was in fact least likely of all the objects to be tested (e.g., simulating changing prioritization from the former most appealing apple to a new, more appealing apple). This condition created an “erased” object (e.g., the original best candidate apple).

Across three experiments, Maxcey-Richard and Hollingworth (2013) found that memory for the erased item was not reliably different from memory for objects that were never cued. In contrast, memory for the cued object on trials with only one cue and memory for the most recently cued object on trials with two cues were better remembered than all the other objects. These behavioral results indicate that when an object is rendered irrelevant, visual working memory for that object returns to baseline, despite its previous status as the prioritized object. Now we will discuss neurophysiological evidence that also indicates that subjects can erase working memory representations when cued to do so.

Williams and Woodman (2012) directed subjects to forget a subset of a memory array while examining an event-related potential (ERP) component, the contralateral delay activity (CDA), known to index the maintenance of items in visual working memory (Vogel and Machizawa, 2004; Vogel et al., 2005; Woodman and Vogel, 2008). As shown in **Figure 1**, subjects in their study were presented with six colored squares, drawn from a set of seven colors, three in each hemifield. After a delay, subjects were cued to forget one lateralized group of the array upon presentation of the words “LEFT” or “RIGHT” on the screen 250 ms into the 2200 ms retention interval. The authors found that instructing subjects to forget the items in one hemifield appeared to eliminate the CDA indexing those forgotten items, as would be expected if the items were erased from visual working memory and only the remaining items were maintained. In a subsequent study, Williams and colleagues (Williams et al., 2013) sought to test the proposal that the items that subjects were cued to forget were in fact erased from memory. They did this by including a handful of trials that probed subjects’ memory for the items they were cued to forget. Using a cued recall procedure (e.g., Zhang and Luck, 2008), they found that subjects had essentially no information in memory about the representations they were cued to forget. These findings clearly suggest that the contents of working memory change based on cues that certain items can be forgotten. However, these findings do not rule out the possibility that information about the items erased from working memory remains in long-term memory.

**FIGURE 1:**
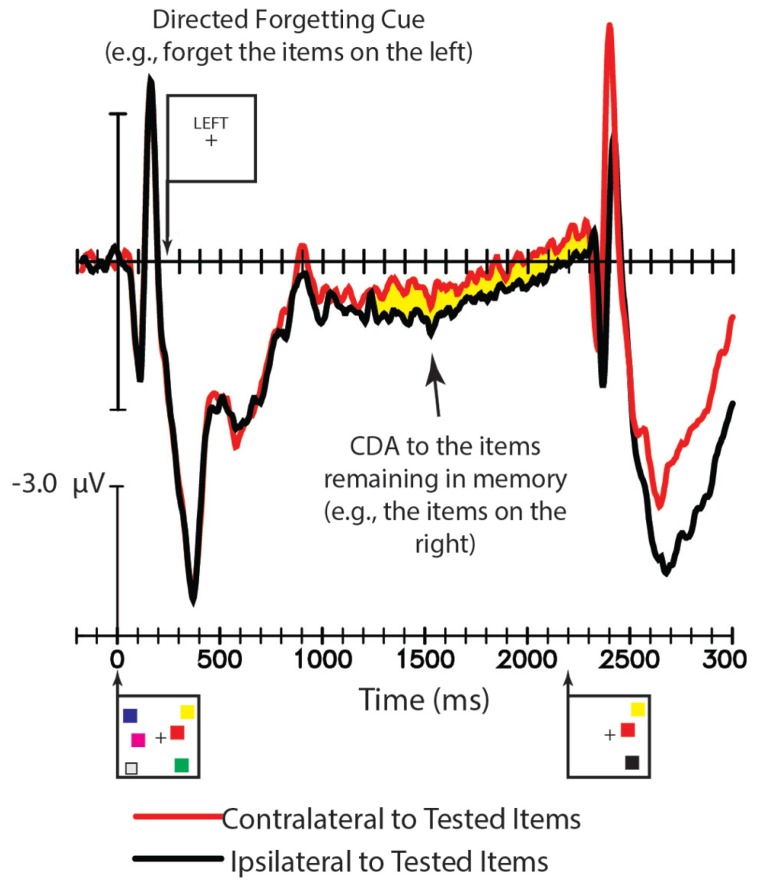
**A portion of the ERP results from from Williams and Woodman (2012) Experiment 3, Figure 8.** The waveforms from T5/6 relative to the tested items on trials with a directed forgetting cue. When subjects were cued to forget all the objects in one hemifield, a CDA emerged. Adapted from “Directed forgetting and directed remembering in visual working memory” by Williams and Woodman (2012; Adapted with permission).

Although these papers concluded that subjects can successfully erase information from working memory, these conclusions hinge on the tasks purely tapping the process of strategically managing working memory without the contributions of long-term memory. Frequently when we study working memory, we do not consider the process purity of the tasks (but see Lin and Luck, 2012). If we predict what kind of effect long-term memory was to have during the tasks that we reviewed above we can expect it to be one of creating proactive interference in which the long-term memory representations from the previous trials will make it harder to remember what was presented on the current trial (e.g., Underwood, 1957). This is because the experiments we described above used a small set of stimuli and repeated them frequently across trials. Frequent repetitions of a small set of stimuli render long-term memory an unreliable source of information about what appeared on any given trial because the activation of all representations would have roughly the same net value. Therefore, we propose that the superior memory for target objects in the studies reviewed above was due to their storage in working memory. However, if these recent studies had not used a small set of stimuli, then the activation state of objects’ long-term memory representations would have reliably differed in a useful way within and across trials. That is, without proactive interference the state of long-term memory representations is sufficient to guide performance. Indeed, theoretical perspectives have stated that one of the primary roles of working memory may be to overcome proactive interference when it has built up or task demands change (Kane and Engle, 2002; Woodman et al., 2013).

Understanding the relationship between working memory and long-term memory in these erasing paradigms is of central importance to current disagreements over the relationship between these memory systems in general. For example, it is possible that working memory is just the activated portion of long-term memory (Cowan, 1995, 2001, 2005; Oberauer, 2002, 2009). If this is correct, then the mechanism driving the erasure from working memory in the studies we reviewed above may be the same mechanism that prevents information from rising to the level of awareness during long-term memory retrieval (Anderson et al., 1994; Anderson and Green, 2001; Anderson and Levy, 2009).

To simulate the inhibition that one attempts to exert in response to the initial activation of an undesired memory, Anderson and Green (2001) used a think/no-think paradigm. In the think/no-think paradigm, subjects learn word pairings such that they can be presented with one of the paired words and retrieve the second word. Then in the think/no-think phase, they are again presented with one of the paired words but in either a color that suggests they should retrieve the second word or in another color that indicates they should not retrieve the second word. The test of whether subjects are successful at preventing the no-think words from entering conscious awareness is accomplished by comparing memory in a final test phase between think, no-think, and baseline items. The critical finding is that successful suppression of the no-think items during the think/no-think phase is exhibited by poorer recall in this final test phase for the no-think items. It may be the case that this successful suppression is actually accomplished by simply not allowing these items into working memory. In contrast, it is possible that failures to keep long-term memory representations out of working memory could underlie the failures to see evidence for memory suppression in this paradigm (e.g., Bulevich et al., 2006), particularly given that the ability to keep task-irrelevant information out of working memory may underlie differences in capacity limits across individuals (e.g., Vogel et al., 2005). This possibility highlights the importance of understanding the relationship between working and long-term memory and the extent of strategic control we have over this relationship.

The relative contributions of working memory and long-term memory are difficult to determine from behavioral data alone. Indeed, it is sometimes debated whether working memory even exists, or whether a single type of memory underlies behavior of healthy humans and neuropsychological patients (Ranganath and Blumenfeld, 2005). Therefore, we propose that neuroscientific measures are required to establish the relationship between these memory systems and explain the effects described above. Specifically, we argue that two ERP components might be particularly useful in revealing whether it is possible to truly erase a representation from memory altogether. The anterior P1, also known as the P170 (Duarte et al., 2004; Friedman, 2004; Diana et al., 2005; Voss et al., 2010; Reinhart and Woodman, 2013), and the FN400, also known as the frontal old/new effect (Rugg et al., 1998), are elicited by the activation of information retained in long-term memory. The anterior P1 is measured at frontocentral sites and is more positive when a subject correctly identifies an object that is stored in visual long-term memory (Tsivilis et al., 2001). The FN400, measured at mid-frontal sites, is more positive when subjects are presented with familiar objects (Rugg et al., 1998). Both of these frontal long-term memory ERPs have been proposed to operate below the explicit awareness of the subject, providing measures of information storage that can be more sensitive than behavioral measures (Tsivilis et al., 2001; Duarte et al., 2004; Friedman, 2004; Rugg and Curran, 2007; Voss et al., 2010; Paller et al., 2012). This means that it should be possible to determine whether information that subjects attempted to erase, nonetheless leaves the residue of information in memory. Similar hypotheses could be tested in a straightforward way using multivariate pattern analyses in functional magnetic resonance imaging experiments (e.g., Norman et al., 2006). These methods are well suited to determine whether information exists in the brain when out of view (e.g., Polyn et al., 2005), and these same methods could be used to determine if memories linger in the brain even after experimental procedures and behavior suggest they should have been erased. Our hope is that future studies can definitively determine whether representations that have been erased from working memory leave residue in long-term memory by measuring these types of brain activity.

Recall that the Maxcey-Richard and Hollingworth (2013) paradigm drew 6–10 objects on each trial from a total set of 10 token objects (20 total stimuli) and the Williams and Woodman (2012) study used six colored squares on each trial, drawn from a total set of seven color squares. These are precisely the conditions that result in high proactive interference among the representations in long-term memory (Wickelgren, 1966). Indeed, it has been proposed that frequent repetitions of a small set of stimuli are necessary to isolate the contribution of working memory to the performance of a task (Cowan, 2001; Lin and Luck, 2012) rendering long-term memory unreliable on a given trial. This means that the studies we reviewed here may have found evidence for the complete erasure of information from memory because people can control what is stored in working memory, but that the potential contributions from long-term memory were not possible to observe due to a buildup of proactive interference. As we discussed above, evidence that working memory changes based on a cue does not mean that long-term memory would not have a representation of that information in a situation where proactive interference is low. We predict that when stimulus sets repeat frequently, as in the studies we reviewed above, contributions from long-term memory are minimal and erasing representations from working memory results in no behavioral evidence for storage of the representation in any memory store. However, if the long-term memory ERPs were measured during these paradigms, we predict that we would see evidence for the residue of the erased representations in memory, below a level that contributes to subjects’ overt behavior. Moreover, when stimuli do not repeat frequently, we predict that contributions from both long-term memory and working memory can be measured with both ERPs and behavior.

Thus, we propose that answering the question of whether information can be erased from memory may do much more than just answer that question of interest to science fiction readers. Instead, we believe that this line of research may answer long-standing questions about the nature of memory. For example, are there multiple memory stores? Do we have the ability to provide top-down control over the contents of our memories? Is it truly possible for a jury to disregard a line of questioning under the direction of a judge?

## Conflict of Interest Statement

The authors declare that the research was conducted in the absence of any commercial or financial relationships that could be construed as a potential conflict of interest.
